# Global landmark: 2023 marks the worst year for dengue cases with millions infected and thousands of deaths reported

**DOI:** 10.1016/j.ijregi.2024.100459

**Published:** 2024-09-26

**Authors:** Najmul Haider, Mohammad Nayeem Hasan, Joshua Onyango, Md Asaduzzaman

**Affiliations:** 1 School of Life Sciences, Faculty of Natural Sciences, Keele University, Keele, United Kingdom; 2 Department of Statistics, Shahjalal University of Science and Technology, Sylhet, Bangladesh; 3 The Harper and Keele Veterinary School, Keele University, Keele, United Kingdom; 4 Department of Engineering, University of Staffordshire, Stoke-on-Trent ST4 2DE, United Kingdom

**Keywords:** Dengue outbreak, Dengue surveillance, Dengue vaccine, Climate change

## Abstract

**Objectives:**

In 2023, the world experienced the worst dengue virus (DENV) outbreak on record. The study aimed to identify global regions and continents with high burden of dengue in 2023.

**Design:**

We collected data on the number of DENV cases and deaths reported by various countries to the World Health Organization and World Health Organization regional offices. We estimated DENV cases per million population and case fatality ratio (CFR) among the confirmed cases reported by each country.

**Results:**

Overall, in 2023 more than 6.5 million cases and over 6,800 deaths attributed to DENV were recorded globally, marking a historic milestone. Two distinct hotspots of DENV circulation emerged: South America and South and Southeast Asian regions. South America reported the highest number of cases (3,924,992), and 1,946 deaths, with a CFR of 0.05. In Asia, 1,622,405 cases and 3,637 deaths were reported, with a CFR of 0.22. We observed a statistically significant difference in the number of cases and deaths per million across different continents (*P*-value <0.001). However, the CFR did not differ significantly across continents (*P*-value = 0.123).

**Conclusions:**

The increased cases and mortality highlight the urgent need for a comprehensive global approach aimed at DENV infection control, including vaccine development, vector control, public health initiatives, and improved clinical management.

## Highlights

• The world is experiencing the largest dengue outbreak ever.
• In 2023, the first global landmark of 6.5 million cases and >6,800 deaths was reached.
• South America reported the highest number of dengue cases (3.9 million).
• Asia had the highest case fatality ratio (0.22).
• Brazil reported the highest number of cases (3.1 million), and Bangladesh reported the highest number of deaths (1,705).

Dengue fever, a mosquito-borne illness, is caused by four distinct serotypes of the dengue virus (DENV) within the Flaviviridae family. Transmission to humans occurs through the bites of *Aedes aegypti (L.)* and *Aedes albopictus* (Skuse) mosquitoes. Currently, DENV is endemic in over 125 countries, with reported cases to the World Health Organization (WHO) escalating annually. Although the majority of infections (>80%) exhibit no or mild symptoms leading to lifelong immunity against the specific serotype, reinfection with different serotypes, termed as secondary dengue infection, poses a significant risk of severe dengue, culminating in fatal outcomes [1].

We collected data from countries where the WHO aids in outbreak confirmation, offers technical support for dengue management, and helps improve reporting systems to accurately capture the disease burden [1]. The WHO recommends several tests for diagnosing dengue infections, including hemagglutination-inhibition, complement fixation, neutralization test, and immunoglobulin M-capture enzyme-linked immunosorbent assay. In addition, some countries use nonstructural protein 1 antigen test for DENV. The details of the laboratory test are discussed elsewhere [2]. Our data sources were from WHO's global dengue surveillance dashboard [3], WHO Eastern Mediterranean Region, WHO European Region [4], WHO Region of Africa, WHO Region of the Americas, WHO South-East Asia Region [5], and WHO Western Pacific Region. We used the national data to accumulate cases by country, continent, and globally. We estimated the cases per million population by countries and continents. A one-way analysis of variance was employed to determine whether dengue variables differ significantly across geographic regions. The case fatality rate of dengue was estimated by dividing the number of deaths by the number of confirmed cases reported by each country.

Globally, a total of 6.43 million cases and 6,892 deaths were recorded in 2023, with 56,672 cases and 28.45 deaths per million population [3]. Continent-wise, the highest number of cases were reported from South America (3,924,992 cases), with the highest number of deaths in Asia (3,637 deaths). The highest number of cases (258,252.27) and deaths (90.30) per million population was reported in North America (Table 1). A one-way ANOVA revealed a statistically significant difference in case incidence and death rates per million across the continents (*P* <0.001), indicating a substantial influence of geographic location on DENV variations. However, when analysing the case fatality rate (CFR) with the same method, the results indicated that CFR did not significantly differ across continents (*P* = 0.123).

**Table 1:** Number of dengue cases and deaths per million (/M) population by continents in 2023.

Continent	Cases	Deaths	Cases/M	*P*-value	Deaths/M	*P*-value	Case fatality ratio (%)	*P*-value
Africa	194,032	832	9,088.38	<0.001	33.63	<0.001	0.43	0.123
Antarctica	0	0	0.00		0.00		0.00	
Asia	1,622,405	3,637	34,416.06		36.03		0.22	
Europe	128	0	2.12		0.00		0.00	
North America	692,109	477	258,252.27		90.30		0.07	
Oceania	1,032	0	69.41		0.00		0.00	
South America	3,924,992	1,946	94,877.55		39.18		0.05	
Total/ average	**6,434,698**	**6,892**	**56,672.26**		**28.45**		**0.11**	

Two distinct hotspots of DENV circulation emerged: the South American and the South and Southeast Asian regions (Table 1). The top five countries with cases of DENV infections in 2023 were Brazil (3,088,723), Vietnam (369,000), Bangladesh (321,179), Mexico (277,963), and Peru (274,227). Brazil reported the highest number of cases (3,088,723), whereas Bangladesh reported the highest number of deaths (1,705) [3,6]. In addition, three European countries reported locally transmitted dengue cases in 2023: Italy (82 cases), France (43 cases), and Spain (three cases), whereas the United States documented a record of 156 locally transmitted cases of DENV [7] (Figure S1 and Table S1).

In the first half of 2024, laboratory-confirmed dengue cases surged to nearly 4.7 million, with over 5,366 deaths being reported globally [3]. With the ever changing summer and rainy seasons across the globe, countries, especially those in the Northern Hemisphere will be bracing for another potentially record-breaking year of DENV, eliciting both anticipation and surprise among observers.

The WHO described several drivers for the largest-ever DENV outbreak in 2023 [1]. The impacts of the 2023 El Niño phenomenon and climate change, resulting in rising temperatures, heavy rainfall, and high humidity; fragile health systems strained by the COVID-19 pandemic; and political and financial instabilities in countries experiencing complex humanitarian crises and significant population movements [1]. The changes in rainfall seasonality and the introduction of heterogeneous serotypes contributed to the largest-ever outbreak in Bangladesh [8]. Brazil reported the highest-ever number of DENV cases in 2023 reported by any other country in the world, which was probably linked to climate change and co-circulation of all four serotypes in the country [1].

Dengue is a major global health issue, impacting millions of individuals each year and presenting significant public health challenges. The incidence of dengue is increasingly being reported in rural areas, broadening its geographical and demographic reach. Dengue infection can vary from mild to severe dengue fever, with fatality rates potentially exceeding 1% [9]. The CFR of primary DENV infection is generally low, with an estimated value of 0.01-0.1%, but the CFR could reach up to 1-4% for secondary or tertiary DENV infection [9]. Before 2023, the highest historic dengue caseload occurred in 2019, with over 3.18 million cases, 28,208 severe cases, and 1,823 deaths (CFR 0.06%) [4]. In 2023, in the South-East Asia Region, Bangladesh observed a rise in deaths from 281 (CFR 0.45%) to 1,705 (CFR 0.52%), whereas Thailand's death toll increased from 34 (CFR 0.07%) to 147 (CFR 0.11%). Other countries reported CFRs ranging from 0.04% in Nepal to 0.72% in Indonesia. In the Western Pacific Region, the Philippines reported 167,355 cases and 575 deaths (CFR 0.34%), while Vietnam reported 149,557 cases and 36 deaths (CFR 0.02%) [4]. Dengue case fatality rates are negatively associated with average income per capita. In addition, primary health care units are linked to lower case fatality rates. A positive association was found between dengue mortality and the Gini index. Overall, investigations into the spatial distribution of dengue incidence indicate that these factors are geographically distinct [10]. The direct costs of dengue, including hospitalization, outpatient visits, and supportive care, are substantial. The indirect costs, such as loss of productivity, long-term disability, and economic losses due to disease outbreaks, are also very high. Furthermore, the disease places a heavy burden on health care systems, resulting in significant economic and social strain.

There were discrepancies between our collected data (6.43 million cases and 6,892 deaths) and the WHO reported 6.5 million cases and over 7,300 dengue-related deaths on their official webpage [1]. We searched the data on WHO's Global Dengue Surveillance dashboard [3] and found no inconsistencies with our findings. However, the differences could have been attributed to variations in case definitions used by different countries. The reported number of cases is likely a significant underestimation of the actual number, as many cases are asymptomatic and do not seek hospital or clinic testing [1]. A study in India found the actual number of cases to be 282 times higher than the reported cases [11]. In Africa, there are fewer reports on dengue and other arboviruses, possibly due to the high burden of malaria, which exhausts most resources [12]. Nevertheless, our findings show that the number of cases and deaths is significant and concerning.

The generalizability of global dengue findings to regions with distinct epidemiologic profiles can be limited due to variations in climate, vector species, population immunity, and health infrastructure. Although global studies provide valuable insights, local factors such as mosquito abundance, behavior, urbanization patterns, and public health responses can significantly influence disease transmission. Therefore, findings from one region may not fully apply to another, highlighting the need for region-specific research and tailored public health interventions to address the unique epidemiologic context of each area [13].

Dengue and other *Aedes*-borne diseases are a critical global health challenge that demands coordinated efforts from multiple sectors. The adaptability of mosquitoes to various breeding sites, including urban environments, and their resistance to insecticides continue to hinder vector control efforts. In addition, although the development of dengue vaccines, such as Dengvaxia and TAK-003, marks significant progress, there are still concerns regarding their efficacy and safety across different age groups and serotypes. Therefore, vaccination strategies must be carefully tailored to specific epidemiologic contexts. To control the ongoing trend of dengue cases, it is essential to enhance epidemiologic surveillance, community engagement and education, environmental management, rapid response to outbreaks, international collaboration, and sustained investment in public health infrastructure, including vaccine development and delivery [14].

## Declarations of competing interest

The authors have no competing interests to declare.

## Appendix Supplementary materials

Image, application 1
