# Supplementary material for: Global landmark: 2023 marks the worst year for dengue cases with millions infected and thousands of deaths reported
Source: IJID Reg. 2024 Sep 26;13:100459. doi: 10.1016/j.ijregi.2024.100459 (PMC11532885; doi:10.1016/j.ijregi.2024.100459)
Supplement: Supplementary file 1 [file mmc1.docx]

**Appendix Figure S1 and Table S1**


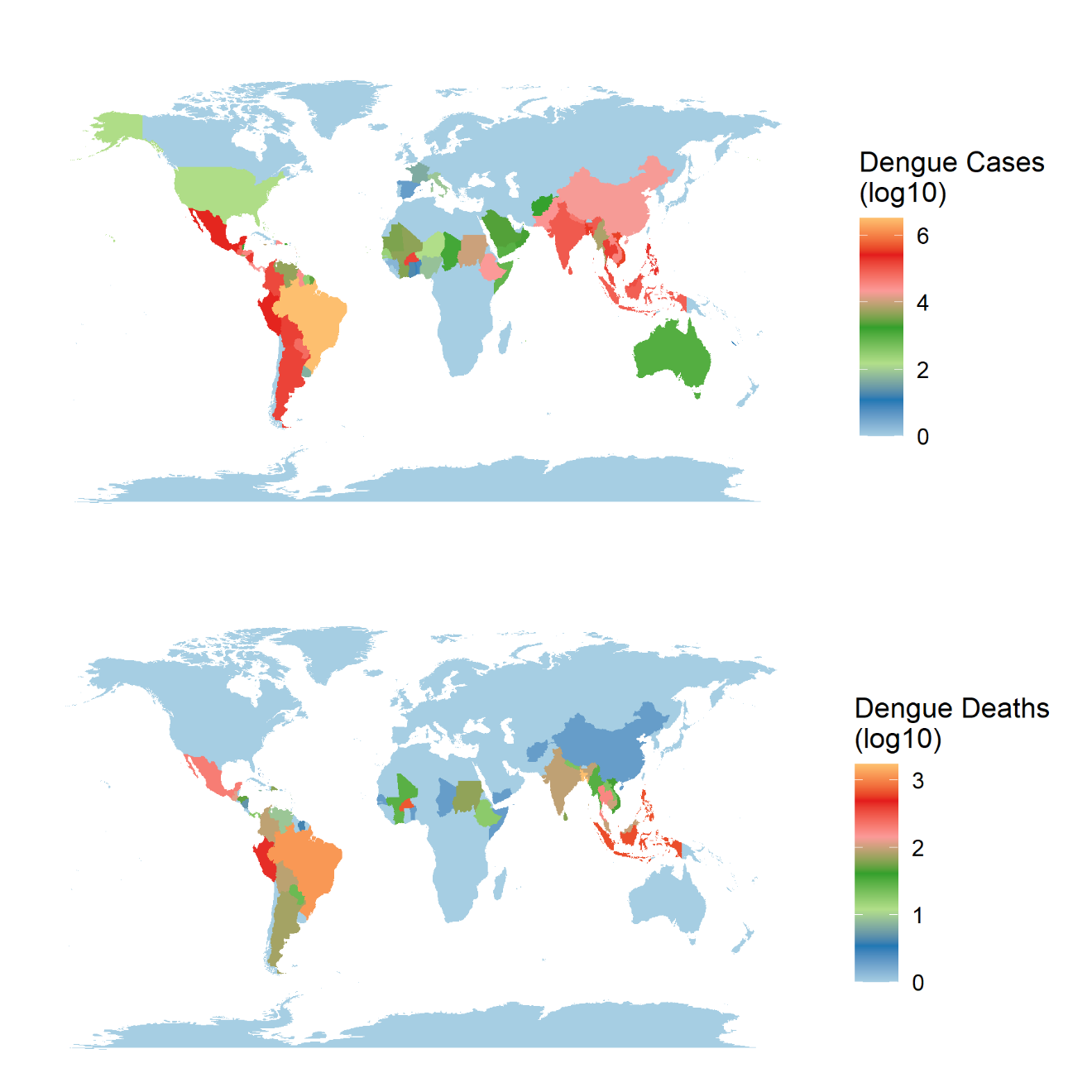


**Figure S1: Number of dengue cases and deaths per million population by country in 2023. Source of the data: WHO global monitoring of dengue** [[3](#ref3)]

**Table S1: Worldwide dengue cases and deaths in 2023. Sources: ECDC, WHO Africa, and WHO** [[3](#ref3)]

| **Country** | **WHO Region** | **Continents** | **Cases** | **Deaths** | **Cases/Million** | **Deaths/Million** |
| --- | --- | --- | --- | --- | --- | --- |
| Afghanistan | WHO Eastern Mediterranean Region | Asia | 1,700 | 1 | 40.25 | 0.02 |
| Oman | WHO Eastern Mediterranean Region | Asia | 2,016 | 0 | 434.07 | 0.00 |
| Pakistan | WHO Eastern Mediterranean Region | Asia | 24,352 | 0 | 101.26 | 0.00 |
| Saudi Arabia | WHO Eastern Mediterranean Region | Asia | 2,259 | 0 | 61.14 | 0.00 |
| Somalia | WHO Eastern Mediterranean Region | Africa | 755 | 1 | 41.61 | 0.06 |
| Sudan | WHO Eastern Mediterranean Region | Africa | 10,412 | 66 | 216.43 | 1.37 |
| Yemen | WHO Eastern Mediterranean Region | Asia | 951 | 1 | 27.61 | 0.03 |
| France | WHO European Region | Europe | 43 | 0 | 0.66 | 0.00 |
| Italy | WHO European Region | Europe | 82 | 0 | 1.39 | 0.00 |
| Spain | WHO European Region | Europe | 3 | 0 | 0.06 | 0.00 |
| Benin | WHO Region of Africa | Africa | 6 | 1 | 0.44 | 0.07 |
| Burkina Faso | WHO Region of Africa | Africa | 146,878 | 688 | 6,316.93 | 29.59 |
| Cape Verde | WHO Region of Africa | Africa | 410 | 0 | 684.84 | 0.00 |
| Chad | WHO Region of Africa | Africa | 1,342 | 1 | 73.42 | 0.05 |
| Ethiopia | WHO Region of Africa | Africa | 21,469 | 17 | 169.68 | 0.13 |
| Ghana | WHO Region of Africa | Africa | 18 | 0 | 0.53 | 0.00 |
| Guinea | WHO Region of Africa | Africa | 1 | 0 | 0.07 | 0.00 |
| Ivory Coast | WHO Region of Africa | Africa | 3,922 | 27 | 135.84 | 0.94 |
| Mali | WHO Region of Africa | Africa | 4,427 | 29 | 190.05 | 1.24 |
| Mauritania | WHO Region of Africa | Africa | 3,582 | 0 | 736.58 | 0.00 |
| Mauritius | WHO Region of Africa | Africa | 265 | 0 | 203.76 | 0.00 |
| Niger | WHO Region of Africa | Africa | 148 | 0 | 5.44 | 0.00 |
| Nigeria | WHO Region of Africa | Africa | 72 | 0 | 0.32 | 0.00 |
| Sao Tome and Principe | WHO Region of Africa | Africa | 69 | 0 | 297.60 | 0.00 |
| Senegal | WHO Region of Africa | Africa | 248 | 1 | 13.96 | 0.06 |
| Togo | WHO Region of Africa | Africa | 8 | 1 | 0.88 | 0.11 |
| Anguilla | WHO Region of the Americas | North America | 1 | 0 | 62.90 | 0.00 |
| Antigua | WHO Region of the Americas | North America | 254 | 0 | 2,693.59 | 0.00 |
| Argentina | WHO Region of the Americas | South America | 146,876 | 75 | 3,208.73 | 1.64 |
| Aruba | WHO Region of the Americas | South America | 22 | 0 | 207.01 | 0.00 |
| Bahamas | WHO Region of the Americas | North America | 243 | 1 | 588.92 | 2.42 |
| Barbados | WHO Region of the Americas | North America | 771 | 0 | 2,734.09 | 0.00 |
| Belize | WHO Region of the Americas | North America | 1,688 | 0 | 4,108.81 | 0.00 |
| Bermuda | WHO Region of the Americas | North America | 1 | 0 | 15.61 | 0.00 |
| Bolivia | WHO Region of the Americas | South America | 156,774 | 88 | 12,654.73 | 7.10 |
| Brazil | WHO Region of the Americas | South America | 3,088,723 | 1,184 | 14,271.73 | 5.47 |
| Cayman Islands | WHO Region of the Americas | North America | 42 | 0 | 605.97 | 0.00 |
| Colombia | WHO Region of the Americas | South America | 131,784 | 90 | 2,530.16 | 1.73 |
| Costa Rica | WHO Region of the Americas | North America | 30,649 | 0 | 5,880.27 | 0.00 |
| Dominica | WHO Region of the Americas | North America | 419 | 0 | 5,736.58 | 0.00 |
| Dominican Republic | WHO Region of the Americas | North America | 27,972 | 62 | 2,468.20 | 5.47 |
| Ecuador | WHO Region of the Americas | South America | 27,838 | 33 | 1,530.36 | 1.81 |
| El Salvador | WHO Region of the Americas | North America | 5,788 | 0 | 909.36 | 0.00 |
| French Guiana | WHO Region of the Americas | South America | 2,684 | 0 | 8,598.29 | 0.00 |
| Grenada | WHO Region of the Americas | North America | 628 | 1 | 4,976.90 | 7.92 |
| Guadeloupe | WHO Region of the Americas | North America | 11,751 | 7 | 29,686.31 | 17.68 |
| Guatemala | WHO Region of the Americas | North America | 72,358 | 119 | 3,999.44 | 6.58 |
| Guyana | WHO Region of the Americas | South America | 27,709 | 0 | 34,047.48 | 0.00 |
| Honduras | WHO Region of the Americas | North America | 34,050 | 49 | 3,214.14 | 4.63 |
| Jamaica | WHO Region of the Americas | North America | 8,180 | 6 | 2,895.02 | 2.12 |
| Martinique | WHO Region of the Americas | North America | 13,239 | 6 | 36,075.44 | 16.35 |
| Mexico | WHO Region of the Americas | North America | 277,963 | 203 | 2,163.88 | 1.58 |
| Montserrat | WHO Region of the Americas | North America | 6 | 0 | 1,367.99 | 0.00 |
| Nicaragua | WHO Region of the Americas | North America | 181,096 | 4 | 25,700.83 | 0.57 |
| Panama | WHO Region of the Americas | North America | 20,924 | 18 | 4,682.99 | 4.03 |
| Paraguay | WHO Region of the Americas | South America | 63,216 | 24 | 9,213.11 | 3.50 |
| Peru | WHO Region of the Americas | South America | 274,227 | 441 | 7,982.69 | 12.84 |
| Puerto Rico | WHO Region of the Americas | North America | 1,242 | 0 | 380.94 | 0.00 |
| Saint Barthelemy | WHO Region of the Americas | North America | 737 | 0 | 67,036.57 | 0.00 |
| Saint Kitts | WHO Region of the Americas | North America | 286 | 1 | 5,988.90 | 20.94 |
| Saint Lucia | WHO Region of the Americas | North America | 60 | 0 | 332.87 | 0.00 |
| Saint Martin | WHO Region of the Americas | North America | 1,272 | 0 | 39,654.58 | 0.00 |
| Saint Vincent | WHO Region of the Americas | North America | 17 | 0 | 163.94 | 0.00 |
| Sint Maarten | WHO Region of the Americas | North America | 1 | 0 | 22.61 | 0.00 |
| Suriname | WHO Region of the Americas | South America | 282 | 3 | 452.48 | 4.81 |
| Trinidad | WHO Region of the Americas | North America | 126 | 0 | 82.09 | 0.00 |
| Turks and Caicos Islands | WHO Region of the Americas | North America | 182 | 0 | 3,951.20 | 0.00 |
| Uruguay | WHO Region of the Americas | South America | 48 | 0 | 14.02 | 0.00 |
| USA | WHO Region of the Americas | North America | 156 | 0 | 0.46 | 0.00 |
| Venezuela | WHO Region of the Americas | South America | 4,809 | 8 | 166.76 | 0.28 |
| Virgin Islands | WHO Region of the Americas | North America | 7 | 0 | 70.89 | 0.00 |
| Bangladesh | WHO South-East Asia Region | Asia | 321,179 | 1,705 | 1,857.02 | 9.86 |
| India | WHO South-East Asia Region | Asia | 94,198 | 91 | 65.94 | 0.06 |
| Maldives | WHO South-East Asia Region | Asia | 3,417 | 0 | 6,558.28 | 0.00 |
| Nepal | WHO South-East Asia Region | Asia | 51,243 | 20 | 1,658.53 | 0.65 |
| Sri Lanka | WHO South-East Asia Region | Asia | 89,799 | 61 | 4,101.61 | 2.79 |
| Thailand | WHO South-East Asia Region | Asia | 159,219 | 179 | 2,217.50 | 2.49 |
| Australia | WHO Western Pacific Region | Oceania | 1,023 | 0 | 38.69 | 0.00 |
| Cambodia | WHO Western Pacific Region | Asia | 35,390 | 99 | 2,088.54 | 5.84 |
| China | WHO Western Pacific Region | Asia | 19,627 | 1 | 13.77 | 0.00 |
| Indonesia | WHO Western Pacific Region | Asia | 83,302 | 633 | 300.15 | 2.28 |
| Laos | WHO Western Pacific Region | Asia | 32,109 | 20 | 4,206.17 | 2.62 |
| Malaysia | WHO Western Pacific Region | Asia | 120,418 | 96 | 3,509.86 | 2.80 |
| Myanmar | WHO Western Pacific Region | Asia | 6,685 | 30 | 122.49 | 0.55 |
| New Caledonia | WHO Western Pacific Region | Oceania | 9 | 0 | 30.72 | 0.00 |
| Philippines | WHO Western Pacific Region | Asia | 195,603 | 657 | 1,667.01 | 5.60 |
| Singapore | WHO Western Pacific Region | Asia | 9,938 | 0 | 1,652.28 | 0.00 |
| Vietnam | WHO Western Pacific Region | Asia | 369,000 | 43 | 3,732.59 | 0.43 |
